# iXora: exact haplotype inferencing and trait association

**DOI:** 10.1186/1471-2156-14-48

**Published:** 2013-06-06

**Authors:** Filippo Utro, Niina Haiminen, Donald Livingstone, Omar E Cornejo, Stefan Royaert, Raymond J Schnell, Juan Carlos Motamayor, David N Kuhn, Laxmi Parida

**Affiliations:** 1 Computational Biology Center, IBM T J Watson Research, Yorktown Heights, NY, USA; 2 USDA-ARS SHRS, Miami, FL, USA; 3 Stanford University, Stanford, CA, USA; 4 MARS, Incorporated, Miami, FL, USA

**Keywords:** Haplotype, Phasing, Phenotype Association, Trait Association, QTL, Randomization

## Abstract

**Background:**

We address the task of extracting accurate haplotypes from genotype data of individuals of large F_1_ populations for mapping studies. While methods for inferring parental haplotype assignments on large F_1_ populations exist in theory, these approaches do not work in practice at high levels of accuracy.

**Results:**

We have designed iXora (Identifying crossovers and recombining alleles), a robust method for extracting reliable haplotypes of a mapping population, as well as parental haplotypes, that runs in linear time. Each allele in the progeny is assigned not just to a parent, but more precisely to a haplotype inherited from the parent. iXora shows an improvement of at least 15% in accuracy over similar systems in literature. Furthermore, iXora provides an easy-to-use, comprehensive environment for association studies and hypothesis checking in populations of related individuals.

**Conclusions:**

iXora provides detailed resolution in parental inheritance, along with the capability of handling very large populations, which allows for accurate haplotype extraction and trait association. iXora is available for non-commercial use from http://researcher.ibm.com/project/3430.

## Background

We address the task of extracting accurate haplotypes from genotype data of individuals of large F_1_ populations for mapping studies. Haplotypes are useful for inferring the underlying causal genetic basis of the traits in mapping populations as one can more efficiently evaluate the parental inheritance of the haplotype implicated in the determination of the trait [1,2]. iXora is specifically suited to plant (or animal) breeding, in which mapping populations of individuals of inbred (or non-inbred) parents are utilized. iXora uses a novel approach by effectively utilizing the large data size and exploiting the fortuitous combinatorial structure in the problem. The algorithm is outlined in the next section with a running example and the mathematical details are presented in Methods.

Given genotypes of *n* progeny on *m* loci, the general problem of constructing haplotypes from genotypes is NP-complete, under various models such as parsimony, maximum likelihood, phylogeny (see [3] for a detailed exposition). Both statistical and combinatorial frameworks have been used in the literature to solve the problem of haplotype extraction from genotype data. For instance, BEAGLE [4,5], fastPHASE [6], HAPI [7], HAPI-UR [8], MACH [9], and SHAPEIT [10,11] use a Hidden Markov Model; whereas Gusfield [12] proposes a combinatorial approach that is based on the parsimony principle. Merlin [13] uses pedigree data under a parsimony model to construct the haplotypes of F_1_ progeny based on their genotypes and the genotypes of the parents. A review of phasing methods, particularly applicable on human data, is presented in [14]. Based on the models of the input population, the existing methods can be categorized into the following scenarios: *unrelated individuals*, *unrelated trios* (two parents per one progeny), and *related trios* (two parents per several progeny, our F_1_ population of interest). These categories are discussed in more detail in Methods.

We compare and contrast iXora with existing phasing programs in literature, summarizing the results in Table 1. The results are described in the Section “Comparison with related methods”. The existing methods are unable to take advantage of the availability of large F_1_ population data without fragmenting it. Through simulation studies we show an improvement of about 15% accuracy in parental haplotype assignment over the next best method. Moreover, iXora runs in linear time and is robust enough to give the same level of accuracy even when the marker data of parents is completely absent.

**Table 1:** Definition and size of the classes

	**Accuracy %**			**Trait**	**Time**	
	**PA (ua)**	**Imp. (ua)**	**PHA %**	**Assoc.**	**Sec**	**Remarks**
	**Unrelated individuals (no parent information)**					
fastPHASE [6]	60.07(0.00)	59.77(0.00)	**NA**	No	78	
	58.01(0.00)	56.55(0.00)			158	
FMPH [12]	-	-	NA	No	-	Up to 30-100 markers
MACH [9]	52.89 (0.00)	52:16(0:00)	**NA**	Yes	567	
	52.49 (0.00)	50.91 (0.00)			1144	
	**Unrelated trios**					
BEAGLE [5]	99.90 (0.00)	98.61 (0.00)	**NA**	Yes	5	
	99.90 (0.00)	98.28 (0.00)			10	
HAPI-UR [8]	99.69 (0.00)	94.75 (0.00)	**NA**	No	3	
	99.67 (0.00)	94.88 (0.00)			7	
	**Related trios**					
HAPI [7]	90.75 (9.17)	0.00 (100.0)	**83.88**	No	0.1	< 15 progeny/parent
	90:63(9:29)	0:00(100:0)	**83.87**		0.2	
Merlin [13]	70:59(29:38)	69:60(29:47)	**69.74**	Yes	299	< 15 progeny/parent
	64:80(35:18)	63:72(35:09)	**63.81**		604	
SHAPEIT2 [11]	87:20(0:00)	57:61(0:00)	**NA**	No	70	< 50 progeny/parent
	90:46(0:00)	64:05(0:00)			148	
iXora	95:89(4:05)	92:11(7:75))	**95.55**	Yes	0.3	
	95:73(4:21)	91:43(8:40)	**95.43**		0.8	

The first row for each method corresponds to 300 markers while the second to 600 markers. The results are averaged over multiple data sets of 200 individuals. Parental haplotype assignment (PHA) was found to be critical in the task of trait association in [15] and is shown here in bold font. Section Methods describes accuracy and PHA computation in detail. Trait assoc. denotes whether the software allows testing for phenotype association. Time was obtained on a system with 3.0 GHz quad-core processor and 4 GB memory. Abbreviations: PA= Parent assignment; Imp. = Imputation; PHA = Parent haplotype assignment; ua = unassigned; “NA” = computation is outside the scope of the software; “-” = unable to compute on our data sets

iXora provides a user-friendly, easy-to-use comprehensive environment for mapping studies. The analysis framework and user interface are described in Additional file 1: Figures A1 and A2. Its usage is described in the section “Using iXora” in Additional file 1, with a running example, where we also demonstrate that genomic regions can be associated with a phenotype at a much higher resolution with haplotypes than with genotypes. The iXora framework has been successfully applied to real data analysis [15], in which pod color phenotype was localized, with high resolution, to a single locus in the *T. cacao* genome.

## Results and discussion

In this section we outline the main results: the iXora phasing algorithm and its comparison with related methods in literature.

### Outline of the core algorithm

**Figure 1:**
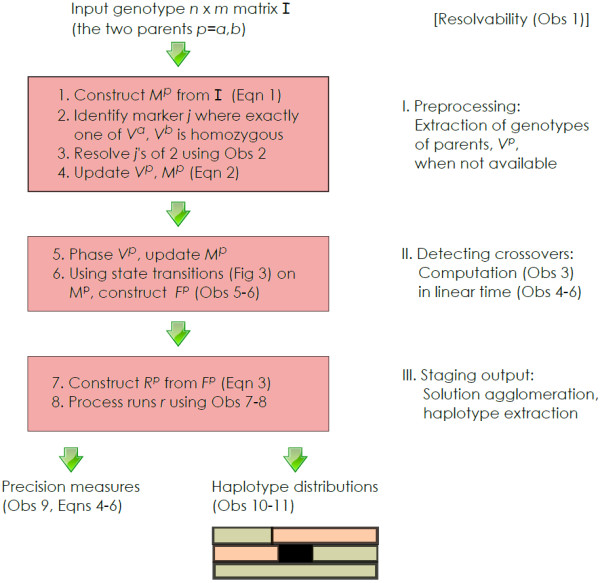
**Outline of the iXora phasing approach.** The eight steps in the iXora haplotype extraction algorithm. Eqn and Obs refer to the Equations and Observations discussed in Methods. The task is to estimate the haplotypes of the two parents, say *a* and *b*, as well as those of the four progeny.

We give an overview of the iXora phasing algorithm here, while the details are described in Methods. The different steps of the algorithm, based on a parsimony principle, are shown in Figure 1. Note that each mathematical observation, with the details in Methods, is abbreviated as *Obs* in the figure and in the description below. To aid in the exposition, we use a concrete example and take the reader through the different steps of the algorithm. The following input progeny genotype matrix,  , is a toy example for illustrative purposes only, with just four individuals (labeled *i*-*iv*) and four bi-allelic markers:

$$I=\left[\begin{array}{l} \text{TC}\\ \text{TT}\\ \text{CC}\\ \text{TC} \end{array}\begin{array}{l} \text{TT}\\ \text{TT}\\ \text{TC}\\ \text{TC} \end{array}\begin{array}{l} \text{AG}\\ \text{AG}\\ \text{GG}\\ \text{GG} \end{array}\begin{array}{l} \text{AG}\\ \text{AG}\\ \text{AG}\\ \text{AG} \end{array}\right]\begin{array}{l} i\\ \text{ii}\\ \text{iii}\\ \text{iv} \end{array}.$$

#### ***Phase I (Preprocessing)***

In Step 1, we initialize two 4 × 4 matrices *M*^*a*^ and *M*^*b*^ based on input . Here a heterozygous position is represented by “X” while a homozygous position is chosen to be 0. Note that if a column has two types of homozygous genotypes, such as TT and CC (as in first column of ), then the first is represented by 0 and the second by 1.

$$\begin{array}{l} i\\ \text{ii}\\ \text{iii}\\ \text{iv} \end{array}\left[\begin{array}{l} X\\ 0\\ 1\\ X \end{array}\begin{array}{l} 0\\ 0\\ X\\ X \end{array}\begin{array}{l} X\\ X\\ 0\\ 0 \end{array}\begin{array}{l} X\\ X\\ X\\ X \end{array}\right]\to M^{a},M^{b}.$$

In this running example, we assume that parent genotypes are missing. That is, the *entire* genotypes of *both* the parents are unavailable. So, in Step 2, we take a first stab at guessing the parents’ haplotypes, labeled *V*^*a*^ and *V*^*b*^, each with two haplotypes indexed as 0 and 1. Since the first marker (*j* = 1) has two types of homozygous genotypes (i.e., TT and CC), it is assumed to be heterozygous in both parents. Also since TT is encoded as 0 in Step 1, the corresponding parent haplotype (index 0) in $V_{1}^{a}$ and $V_{1}^{b}$ is T, i.e., $V_{1}^{a}(0)=V_{1}^{b}(0)=T$. CC is encoded as 1, thus $V_{1}^{a}(1)=V_{1}^{b}(1)=C$. The second marker (*j* = 2) has only one homozygous genotype (i.e., TT), and is assumed to be homozygous in only one of the parents. Similarly, the third marker (*j* = 3) is homozygous in only one of the parents, but the fourth (*j* = 4) marker is assumed to be homozygous in both parents. Thus only the second and the third marker need to be resolved further and this is summarized as follows (currently unresolved values denoted by “?” and homozygous by “H”):

$$\begin{array}{l} 0\\ 1 \end{array}\left[\begin{array}{l} \text{T}\\ \text{C} \end{array}\begin{array}{l} \text{?} \end{array}\begin{array}{l} \text{?} \end{array}\begin{array}{l} \text{H} \end{array}\right]\to V^{a},V^{b}.$$

Without loss of generality, let the second marker be homozygous in parent *b*. The third marker is resolved relative to the second marker based on a global *polarization* rule (Obs 2 in Methods). This heuristic works on column 2 (*j*) and column 3 ($j^{\prime }$) to compute $C_{jj^{\prime }}$ that tracks the four counts corresponding to the four haplotype pairs 00, 01, 10 and 11 labeled as *c*_00_, *c*_01_, *c*_10_ in *c*_11_ respectively (count *c*_*xy*_ is the number of individuals where marker at *j* is inherited from haplotype *x* and the marker at *j*^′^ is inherited from haplotype *y* of the same parent). In this rather simple example, all the four counts are zero, and thus $C_{jj^{\prime }}$ is not polarized. Then the second and third markers are homozygous in *different* parents. Hence the third marker is homozygous in parent *a*. This constitutes Step 3 and the results are encoded in the two parents as follows (here “X” denotes heterozygous, “H” denotes homozygous locus and “-” denotes homozygous in both parents):

$$\begin{array}{l} V^{a}=\left[\begin{array}{l} T\\ C \end{array}\begin{array}{l} X \end{array}\begin{array}{l} H \end{array}\begin{array}{l} H \end{array}\right]=\left[\begin{array}{l} T\\ C \end{array}\begin{array}{l} T\\ C \end{array}\begin{array}{l} G\\ G \end{array}\begin{array}{l} \text{-}\\ \text{-} \end{array}\right]\begin{array}{l} 0\\ 1 \end{array} \end{array}$$

and

$$\begin{array}{l} V^{b}=\left[\begin{array}{l} T\\ C \end{array}\begin{array}{l} H \end{array}\begin{array}{l} X \end{array}\begin{array}{l} H \end{array}\right]=\left[\begin{array}{l} T\\ C \end{array}\begin{array}{l} T\\ T \end{array}\begin{array}{l} G\\ A \end{array}\begin{array}{l} \text{-}\\ \text{-} \end{array}\right]\begin{array}{l} 0\\ 1 \end{array}. \end{array}$$

With these assignments of the parent haplotypes, the progeny haplotype assignment matrices *M*^*a*^ and *M*^*b*^ are updated in Step 4 as shown below. Note that, if the genotypes of the parents are available then Steps 2-3 are redundant and *V*^*a*^, *V*^*b*^ are initialized directly using the input genotypes of the parents.

$$M^{a}=\left[\begin{array}{l} X\\ 0\\ 1\\ X \end{array}\begin{array}{l} 0\\ 0\\ 1\\ 1 \end{array}\begin{array}{l} H\\ H\\ H\\ H \end{array}\begin{array}{l} H\\ H\\ H\\ H \end{array}\right]\begin{array}{l} i\\ \text{ii}\\ \text{iii}\\ \text{iv} \end{array},M^{b}=\left[\begin{array}{l} X\\ 0\\ 1\\ X \end{array}\begin{array}{l} H\\ H\\ H\\ H \end{array}\begin{array}{l} 1\\ 1\\ 0\\ 0 \end{array}\begin{array}{l} H\\ H\\ H\\ H \end{array}\right]\begin{array}{l} i\\ \text{ii}\\ \text{iii}\\ \text{iv} \end{array}.$$

#### ***Phase II (Detecting crossovers)***

A marker *j* is homogenous in matrix *M*^*p*^ if the entire column *j* in *M*^*p*^ is marked as *H*. In Step 5, the polarization rule is applied again to pairs of adjacent non-homogenous markers (*j* and *j*_*nxt*_) in each of the matrices *M*^*a*^ and *M*^*b*^ separately. These result in possible switching of marker values in the parent haplotypes *V*^*p*^ and the progeny at that marker in *M*^*p*^. The reader will observe that no switching is made in *M*^*a*^. However, in *M*^*b*^, for *j* = 1 and *j*_*nxt*_ = 3, the four counts are *c*_01_ = 1, *c*_10_ = 1 and *c*_00_ = *c*_11_ = 0. Since $\left(c_{01}+c_{10})>(c_{00}+c_{11}\right)$, marker *j*_*nxt*_ is switched in *V*^*b*^ and *M*^*b*^ to yield the following:

$$\begin{array}{lll} V^{a} & = & \left[\begin{array}{l} T\\ C \end{array}\begin{array}{l} T\\ C \end{array}\begin{array}{l} G\\ G \end{array}\begin{array}{l} \text{-}\\ \text{-} \end{array}\right]\begin{array}{l} 0\\ 1 \end{array},\\ M^{a} & = & \left[\begin{array}{l} X\\ 0\\ 1\\ X \end{array}\begin{array}{l} 0\\ 0\\ 1\\ 1 \end{array}\begin{array}{l} H\\ H\\ H\\ H \end{array}\begin{array}{l} H\\ H\\ H\\ H \end{array}\right]\begin{array}{l} i\\ \text{ii}\\ \text{iii}\\ \text{iv} \end{array}, \end{array}\begin{array}{lll} V^{b} & = & \left[\begin{array}{l} \text{T}\\ \text{C} \end{array}\begin{array}{l} T\\ T \end{array}\begin{array}{l} A\\ G \end{array}\begin{array}{l} \text{-}\\ \text{-} \end{array}\right]\begin{array}{l} 0\\ 1 \end{array},\\ M^{b} & = & \left[\begin{array}{l} X\\ 0\\ 1\\ X \end{array}\begin{array}{l} H\\ H\\ H\\ H \end{array}\begin{array}{l} 0\\ 0\\ 1\\ 1 \end{array}\begin{array}{l} H\\ H\\ H\\ H \end{array}\right]\begin{array}{l} i\\ \text{ii}\\ \text{iii}\\ \text{iv} \end{array}. \end{array}$$

Some systematic transitions (Obs 5-6 in Methods) are applied to the non-numeric elements of the *M*^*a*^ and *M*^*b*^, in Step 6, to obtain the following:

$$F^{a}=\left[\begin{array}{l} e_{0}\\ 0\\ 1\\ e_{1} \end{array}\begin{array}{l} 0\\ 0\\ 1\\ 1 \end{array}\begin{array}{l} e_{0}\\ e_{0}\\ e_{1}\\ e_{1} \end{array}\begin{array}{l} e_{0}\\ e_{0}\\ e_{1}\\ e_{1} \end{array}\right]\begin{array}{l} i\\ \text{ii}\\ \text{iii}\\ \text{iv} \end{array},F^{b}=\left[\begin{array}{l} e_{0}\\ 0\\ 1\\ e_{1} \end{array}\begin{array}{l} e_{0}\\ e_{0}\\ e_{1}\\ e_{1} \end{array}\begin{array}{l} 0\\ 0\\ 1\\ 1 \end{array}\begin{array}{l} e_{0}\\ e_{0}\\ e_{1}\\ e_{1} \end{array}\right]\begin{array}{l} i\\ \text{ii}\\ \text{iii}\\ \text{iv} \end{array}.$$

#### ***Phase III (Staging output)***

In this toy example, we can simply transform *e*_0_ to 0 and *e*_1_ to 1 to obtain the phasing result in *R*^*a*^ and *R*^*b*^ (Steps 7-9): 

The parent haplotypes are encoded in *V*^*a*^, *V*^*b*^ respectively and the progeny haplotypes in *R*^*a*^ and *R*^*b*^. The solution shows no recombinations in the progeny, but has two errors between the phased sequences and the observed genotypes, shown in red. We omit the evaluation of the results (precision measures) here, and direct the reader to Methods.

### Comparison with related methods

Here we describe the results from a simulation study on a F_1_ population with 200 individuals and 300–600 markers. The parameters of the simulation were chosen to reflect real data and the details are described in the section “Using iXora” in Additional file 1.

We compare iXora with the existing phasing methods BEAGLE [5], fastPHASE [6], FMPH [12], HAPI [7], HAPI-UR [8], MACH [9], Merlin [13], and SHAPEIT2 [11]. Each existing method solves a slightly different phasing problem, such as not providing a parental haplotype assignment for the progeny, or not processing the entire population in one run, as discussed in Methods. Hence we used evaluation criteria that enable a meaningful comparison of this wide-spectrum of methods, when applied on simulated data. The evaluation criteria are described in Methods, and the results are summarized in Table 1.

#### ***Accuracy of Parent Assignments (PA)***

This accuracy is measured on a marker-by-marker basis. We post-process the output of the systems that do not directly provide parental assignment. The best parental assignment is seen with BEAGLE and HAPI-UR, followed by iXora. In the two former cases, there are no unassigned markers. HAPI and SHAPEIT2 show moderate accuracy while Merlin, fastPHASE, and MACH perform poorly. Note that Merlin’s performance deteriorates with the increase in number of markers, while HAPI and iXora display similar levels of accuracy.

#### ***Handling missing data/imputation***

All the methods, except HAPI, show some capability of handling missing data. Merlin has about a third of the missing data unresolved, while iXora has less than 10% unresolved. The rest of the methods resolve all the missing data. BEAGLE, HAPI-UR and iXora display levels of accuracy in the imputed data larger than 90% while the rest perform poorly. Note that these values only account for missing data in the progeny. We found that missing data in the parents were debilitating for all the trio based methods, except Merlin and iXora. These two methods were the only ones that produced some results when *all* the marker data of both parents were missing. Since Merlin can handle only a small number of individuals per parent, about 15% of the parent haplotypes remained unresolved. We observed that iXora is the only method robust enough to be unaffected by completely missing parent genotypes. We attribute this resilience to its ability to handle large families of individuals without splitting into smaller sets.

#### ***Accuracy of Parent Haplotype Assignments (PHA)***

Note that PHA is the most important computation since this crucially contributes to the improvement in accuracy and resolution in genomic region assignment to traits (see “Using iXora” in Additional file 1 for an example). In Table 1 the corresponding column, labeled PHA, is shown in bold and is the focus of the comparison study.

The PHA accuracy is measured on a marker-by-marker basis. Only HAPI, Merlin, and iXora provide an assignment of the parental haplotypes. Note that although SHAPEIT2 utilizes trios, it did not give us any means to extract parent haplotype information from the output. Both HAPI and Merlin perform poorly, with accuracy under 85% and 70% respectively. In contrast, iXora yields an accuracy of over 95%.

Although, HAPI and Merlin give means of identifying the parent haplotypes, they suffer a severe scaling problem, and are unable to handle more than about ten progeny per family. Thus it is not obvious how these systems can be coaxed to exploiting the availability of large progeny to improve the accuracy of the parental haplotype assignments.

## Conclusions

From the comparison with related methods, we conclude that while methods to the problem of inferencing parental haplotype assignments on large F_1_ populations exist in theory, these approaches do not work in practice at high levels of accuracy (say > 90%). Moreover, iXora is the only algorithm that is robust enough to accurately extract the parental haplotypes in the absence of any parental genotype information. In practice, when the genotypes of the parents were known, we used this capability of iXora to match the estimated parent genotypes against the true genotypes to confirm the integrity of the phasing results. iXora additionally outputs several intrinsic measures of preciseness (the triplet $\Delta_{I},D_{I},E_{I}$), and all the equally-likely phasing solutions with annotations (q/Q/*), see Methods. These added capabilities make iXora and its output particularly attractive, over existing methods, for trait association and inferencing studies.

## Methods

In this section we describe the mathematical details of the iXora haplotype inference algorithm, the measures used to quantify the precision of the output, and the different downstream processing of the output. We conclude the section with the description of the measures used to compare the results from different phasing algorithms.

### iXora algorithm: haplotype inferencing

The outline of the three phases in the iXora algorithm is shown in Figure 1. In this section what follows is a more precise mathematical description of the steps which is presented as a sequence of key observations: thus this describes as well as provides the rationale for each of the steps. Note that Figure 1, annotated by equation and observation numbers, is a road map for the exposition in this section to help the reader understand the description.

#### ***Notation***

Let  be an *n* × *m* matrix that encodes *n* (> 1) progeny with *m* (> 1) markers. Each row *i* represents a progeny and each column *j* represents a marker. The order of the markers is also captured in the matrix, i.e., $j^{\prime }<j<j^{\prime }{\;}^{\prime }$ if and only if marker *j* is located between markers *j*^′^ and *j*^′^^′^ on the chromosome. The *j*th marker of the *i*th progeny, denoted $I_{\text{ij}}$ or simply 〈*ij*〉, also referred to as the position 〈*ij*〉, is a pair of alleles: each individual allele can be accessed as $I_{\text{ij}}(0)$ and $I_{\text{ij}}(1)$. Thus if $I_{\text{ij}}=\{A,C\}$ or simply written as AC, $I_{\text{ij}}(0)=$ A and $I_{\text{ij}}(1)=$ C. The two observed alleles at marker *j* (across all individuals) will be denoted as *Z* and *Z*^′^. A marker *j* is *polymorphic* if *Z* ≠ *Z*^′^. We make the assumption that *all* the markers in  are polymorphic. When $I_{\text{ij}}=\text{ZZ}$ or $I_{\text{ij}}=Z^{\prime }Z^{\prime }$ the *j*th marker at individual *i*, or position 〈*ij*〉, is said to be *homozygous*. Similarly, when $I_{\text{ij}}=ZZ^{\prime }$, position 〈*ij*〉 is said to be *heterozygous*.

We next introduce a definition and notation for conjugacy. Let *conjugate* of *z* be written as $\tilde{z}$, where *z* is a matrix or a discrete value as defined below. Note that the conjugate of conjugate of *z* is *z*, i.e., $\tilde{\tilde{z}}=z$ always holds. For parents *p* = *a*,*b*, if *p* = *a*, then $\tilde{p}=b$ and vice-versa. Similarly, if *k* = 0, then $\tilde{k}=1$ and vice-versa. Thus $M^{a}={\tilde{M}}^{b}$ (and $M^{b}={\tilde{M}}^{a}$). Also, ${\tilde{V}}^{p}(0)=V^{p}(1)$ and ${\tilde{V}}^{p}(1)=V^{p}(0)$ for the parents *p* = *a*,*b*.

#### ***Solvability of a given genotype matrix***

Assume that there is no more than one crossover, in an individual, between two adjacent positions. If *d*_*t*_ is the true number of such crossovers, then 0≤*d*_*t*_ ≤ 2(*m* - 1)*n*. Let the estimate of *d*_*t*_ be *d*_*c*_ ≥ 0. Consider a scenario where each position in  is heterozygous. Then there exist an exponentially large number (2^*mn*^) of distinct and equally-likely haplotype configurations of the progeny, each with *no* crossovers. While informative markers in general are chosen for their heterozygosity in data sets, it is observed that the same marker is also homozygous in many a progeny. Thus, in practice, due to Mendelian inheritance [16] it is very unlikely to have a run of markers that are heterozygous in all the progeny. It is this random sprinkling of homozygous positions in , that makes *d*_*c*_ estimation possible. We conducted simulations to study the relationship between *d*_*c*_ and the extent of homozygosity in  in realistic data scenarios.

Let *e* denote the mean number of crossovers in a progeny. We used simulations with values 2 ≤ *e* ≤ 15. We observed that for this wide range of crossover profiles, the required fraction of homozygous sites in  to get a good estimate of *d*_*t*_ (i.e., within 5% of the true value) was bound. The empirical observation is summarized below.

##### 

**Observation 1.** *When at least 28% of each subsample of randomly chosen positions in **is homozygous, the estimated d*_*c*_ *is within 5% of the exact value d*_*t*_, i.e., *d*_*c*_ *≥* *0.95d*_*t*_.

In practice, we have encountered values of *n* in the range of 50 to 400 and *m* in the range of 30 to 600. We observe that consistently, at least 50% positions are homozygous and the solutions, obtained using methods of the following sections, displayed *e* ≈ 2. With decrease in cost and in increase in accessibility of sequencing and genotyping technologies, *m* could be orders of magnitude larger in the coming years. Note that *e* is not expected to increase with *m*. Thus the lower bound estimation scales well with *m*, since the observation above is independent of *m* and the algorithm discussed in the later sections is linear in *m*.

#### ***Phase I: Preprocessing***

Since all the individuals have the same two parents, let the two parents be *a* and *b*. Let *V*^*a*^(0) encode haplotype 0 of parent *a* and *V*^*a*^(1) encode haplotype 1 of parent *a*. Similarly, *V*^*b*^(0) and *V*^*b*^(1). The two distinct allele values of parent *p* at marker *j* are represented by $V_{j}^{p}(0)$ and $V_{j}^{p}(1)$.

At Step 1, *M*^*a*^ and *M*^*b*^ are initialized, for each *i* and *j* and *p* = *a*,*b*, as follows. Let the two alleles at marker *j* be written as *Z*_*j*_ and $Z_{j}^{\prime }$.

(1) $$M_{\text{ij}}^{p}=\left\{\begin{array}{ll} 0 & \text{if}\;I_{\text{ij}}=Z_{j}Z_{j},\\ 1 & \text{if}\;I_{\text{ij}}=Z_{j}^{\prime }Z_{j}^{\prime },\\ X & \text{if}\;I_{\text{ij}}=Z_{j}Z_{j}^{\prime }. \end{array}\right)$$

Also, *V*^*a*^ and *V*^*b*^ is initialized as $V_{j}^{p}(0)=Z_{j}$ and $V_{j}^{p}(1)=Z_{j}^{\prime }$, for each *j* and *p* = *a*,*b*.

If marker *j* is homozygous in both parents, then position 〈*i*,*j*〉 is heterozygous, for all progeny *i*. If marker *j* is not homozygous in both parents, then possible progeny values are *Z*_*j*_*Z*_*j*_, $Z_{j}^{\prime }Z_{j}^{\prime }$, and $Z_{j}Z_{j}^{\prime }$. When both parents are homozygous, it may not be apparent what the allelic values of the individual parents are, but as is seen in the running example of the Overview section, this does not affect the solution. Such loci are marked as “-” in the parents, i.e., $V_{j}^{p}(0)=V_{j}^{p}(1)=$“-” for *p* = *a*,*b*. Also the column *j* of the matrices are updated as $M_{\text{ij}}^{a}=M_{\text{ij}}^{b}=H$, for each *i*. If exactly one parent (either *a* or *b*) is homozygous, then only *Z*_*j*_*Z*_*j*_, but not $Z_{j}^{\prime }Z_{j}^{\prime }$, can be observed in some progeny, while the rest are $Z_{j}Z_{j}^{\prime }$. In Step 2, we identify all such markers.

Note that while it is easy to estimate if a marker is homozygous in both parents or heterozygous in both, it is not obvious to estimate the heterozygous parent when exactly one of the parents is so. In Step 3 we identify markers which are homozygous in exactly one parent (i.e., either *a* or *b*). The execution of this process is illustrated in the Overview section through the running example and is described in detail here.

Recall that *a markerj* is homozygous in parent *p* if all the entries in *M*^*p*^ are H. For a fixed *p*, for a pair of non-homozygous markers *j* and *j*^′^ four counts, $c_{k_{1}k_{2}}$ are computed for all combinations of *k*_1_,*k*_2_ ∈ {0,1} as:

$$c_{k_{1}k_{2}}=|\{i|M_{\text{ij}}^{p}=k_{1}\;\text{and}\;M_{ij^{\prime }}^{p}=k_{2},p=a,b\}|.$$

In words, *c*_01_ is the count of individuals where marker at *j* is inherited from haplotype 0 of the parent *p* and the marker at *j*^′^ is inherited from haplotype 1 of the same parent. Similarly, *c*_10_, *c*_11_ and *c*_00_. Let

$$C_{jj^{\prime }}=\{c_{00},c_{11},c_{01},c_{10}\}.$$

Let *t* = *c*_00_ + *c*_11_ and *t*^′^ = *c*_01_ + *c*_10_. Also, let *t*_max_ = max{*t*,*t*^′^} and *t*_min_ = min{*t*,*t*^′^}. Then this is interpreted as *t*_max_ individuals with no crossovers while *t*_min_ individuals have a crossover between locations *j* and *j*^′^. In practice, we use a stricter condition where the values *t*_max_ and *t*_min_ need to be well separated. We use two threshold fractions: 0 < *α*_1_,*α*_2_ ≤ 1. $C_{jj^{\prime }}$ is said to be *polarized* if and only if the following hold:

$$\underset{x\in C_{jj^{\prime }}}{\sum }x\geq n\alpha_{1}\;\text{and}\;\frac{t_{\text{min}}}{t_{\text{max}}}<\alpha_{2}.$$

In our implementation we use *α*_1_ = 0.3 and *α*_2_ = 0.15, which we have observed to yield accurate phasing results on real data, confirmed by the precision measures discussed later. When the polarization condition is violated, it is considered to be an error in data . In practice, we observed that in all such cases this was due to experimental errors at one of the markers. Hence, we make the following assumption. *For parentp* and for the pair of non-homozygous markers *j* and *j*^′^, $C_{jj^{\prime }}$*must be polarized.* In practice, when this condition is violated, we flag the marker for closer scrutiny in the experiments. A consequence of this assumption is the following.

##### 

**Observation 2.** *Let J*_*a*_ *(resp J*_*b*_*) be the set of markers with heterozygous parent a (resp b). If *$C_{jj^{\prime }}$*is not polarized, then j* *∈* *J*_*p*_ *and *$j^{\prime }\in J_{\tilde{p}}$.

This observation states that it is possible to computationally obtain two non-overlapping sets of markers, where one set represents the markers that are homozygous in only parent *a* and the other set represents the markers that are homozygous in only parent *b*. Thus, when the parent genotypes are not available, this observation is utilized in Step 3 to resolve the markers that are homozygous in exactly in one parent.

Let marker *j* be homozygous only in parent *a* based on the above process, then $V_{j}^{p}$ and column *j* in *M*^*a*^ and *M*^*b*^ are updated (Step 4) as follows: $V_{j}^{a}(0),V_{j}^{a}(1),V_{j}^{b}(0)\leftarrow Z_{j}$, and $V_{j}^{b}(1)\leftarrow Z_{j}^{\prime }$. Next, column *j* in *M*^*p*^ is updated as follows:

(2) $$\begin{array}{ll} M_{\text{ij}}^{p} & \leftarrow \left\{\begin{array}{ll} H & \text{if}\;V_{i}^{p}(1)=H,\\ 1 & \text{else if currently}\;M_{\text{ij}}^{p}\;\text{is}\;X,\\ 0 & \text{else if currently}\;M_{\text{ij}}^{p}\;\text{is}\;0. \end{array}\right)\; \end{array}$$

Note that the above is equivalent to: For a marker *j* that is homozygous only in parent *a*: $M_{\text{ij}}^{a}$ is set to *H* for all *i* and if $M_{\text{ij}}^{a}$ was *X*, then it is updated to 1. Similarly, for a marker *j* that is homozygous only in parent *b*: $M_{\text{ij}}^{b}$ is set to *H* for all *i* and if $M_{\text{ij}}^{b}$ was *X*, then it is updated to 1.

*Rationale.* Note parent *a* is homozygous at *j* (but parent *b* is not). The reason for switching *X* to 1 in column *j* of *M*^*b*^ is that no matter what the value of $M_{\text{ij}}^{a}$ is, or will be eventually updated to, 1 encodes for the allele *Z*’.

When marker *j* is homozygous only in parent *b*, a similar update as above is performed. Note that in the implementation, as a marker *j* is resolved (Step 3), the marker is immediately updated in *V*^*p*^ and *M*^*p*^ (Step 4).

#### ***Phase II: Detecting crossovers***

Without loss of generality, for each non-homozygous *j* in *M*^*p*^, let *j*_*nxt*_ be the adjacent non-homozygous marker in *M*^*p*^. Substituting *j*^′^ with *j*_*nxt*_ in the definitions from the last section, let *t*_max_ and *t*_min_ be redefined. Then in Step 5, *V*^*p*^ is phased as follows: For parent *p*, if $t_{\text{max}}=c_{01}+c_{10}$, then the values of $V_{j}^{p}(0)$ and $V_{j}^{p}(1)$ are interchanged. Further, column *j* in *M*^*p*^ is updated by replacing an existing entry of 0 with 1 and an entry of 1 with 0, to reflect the updated $V_{j}^{p}$.

Let $xo^{p}(j,j_{\text{nxt}})=t_{\text{min}}$. Then the minimal number of crossovers (or recombinations) between *j* and *j*_*nxt*_ in haplotypes inherited from parent *p* is $xo^{p}(j,j_{\text{nxt}})$. Thus:

##### 

**Observation 3.** *If d*_*c*_ *is the total number of estimated crossovers, then*

$$\left(\underset{j}{\sum }xo^{a}(j,j_{\text{nxt}})+\underset{j}{\sum }xo^{b}(j,j_{\text{nxt}})\right)\leq d_{c}.$$

Note that *d*_*c*_ could be larger than the sum on the left, since some further crossovers may be introduced in the next phase. Since only adjacent markers need to be considered for detecting crossovers, the following holds.

##### 

**Observation 4.** *Given **, the haplotype matrices M*^*a*^,*M*^*b*^ *and the parent haplotypes V*^*a*^,*V*^*b*^ *can be constructed in *O(mn)*time*.

##### 

**Missing values** There are often missing values in the data, sometimes as high as 20%. When the value is missing at position 〈*ij*〉, we make the assignment $M_{\text{ij}}^{p}\leftarrow X$, for *p* = *a*,*b*. However, if after the computation of *V*, marker *j* is homozygous at parent *p*, then $M_{\text{ij}}^{p}\leftarrow H$ and $M_{\text{ij}}^{\tilde{p}}$ is appropriately updated. It can be verified that this treatment does not introduce any extra (spurious) crossovers.

##### 

**Selfed progeny** For selfed progeny, not only are the parents the same but even the haplotypes of the parents are deemed identical. Then one of the following conditions holds for *p* = *a*,*b* and a numeric *k* ∈ {0,1}: 

(1) *V*^*a*^=*V*^*b*^ and if $M_{\text{ij}}^{p}=k$ then $M_{\text{ij}}^{\tilde{p}}=\tilde{k}$, for all *i* and *j*, or,

(2) *V*^*a*^ ≠ *V*^*b*^ and if $M_{\text{ij}}^{p}=k$ then $M_{\text{ij}}^{\tilde{p}}=k$, for all *i* and *j*.

Thus *M*^*b*^ is defined completely by *M*^*a*^ and thus the task is to estimate only *M*^*a*^.

##### 

**Monotonic state transitions** Next, the matrices *M*^*p*^ are transformed as follows. A position 〈*ij*〉 is a *heterozygous trio*, if the two parents at *j* are heterozygous and so is $I_{\text{ij}}$.

Let *k* represent a numeric value. Define two functions Lt(·) and Rt(·) on an element of the matrix as follows:

$$\begin{array}{lll} \text{Lt}(M_{\text{ij}}) & =\left\{\begin{array}{ll} \text{Rt}(M_{\text{ij}}) & \text{if}j=1,\text{i.e.,jis leftmost,}\\ \;k & \text{else if}M_{ij^{\prime }}=\text{k for}1\leq j^{\prime }<j,\\ \text{and there is no}j^{\prime }<j^{\prime }{\;}^{\prime }<j\\ \text{with numeric}M_{ij^{\prime }{\;}^{\prime }},\\ \;-1\text{otherwise.} \end{array}\right)\; & \\ \text{Rt}(M_{\text{ij}}) & =\left\{\begin{array}{ll} \text{Lt}(M_{\text{ij}}) & \text{if}j=m,\text{i.e.,jis rightmost,}\\ \;k & \text{else if}M_{ij^{\prime }}=k\text{for}j\leq j^{\prime }<m,\\ \text{and there is no}j^{\prime }>j^{\prime }{\;}^{\prime }>j\\ \text{with numeric}M_{ij^{\prime }{\;}^{\prime }},\\ \;-1\text{otherwise.} \end{array}\right)\; & \end{array}$$

Note that a marker *j* can never be both leftmost and rightmost since the number of markers is at least two, hence both Lt(·) and Rt(·) are well defined. Let *M*_*ij*_ = *x*. Then the transition *S*_*x*_→*S*_y_ is applied to assign the value *y* to *M*_*ij*_ (written as *M*_*ij*_←*y*) as follows. 

o $S_{H}\to S_{e_{k}},S_{X}\to S_{e_{k}}$

o If Lt(*M*_*ij*_) = Rt(*M*_*ij*_) = *k*, then *M*_*ij*_ ← *e*_*k*_.

o $S_{H}\to S_{w_{k_{1}k_{2}}}^{H},S_{X}\to S_{w_{k_{1}k_{2}}}^{X}$

o If Lt(*M*_*ij*_) ≠ Rt(*M*_*ij*_) with Lt(*M*_*ij*_) = *k*_1_ and Rt(*M*_*ij*_) = *k*_2_, then $M_{\text{ij}}\leftarrow w_{k_{1}k_{2}}.$

o $S_{w_{k_{1}k_{2}}}^{X}\to S_{e_{k}}$, $S_{w_{k_{1}k_{2}}}^{H}\to S_{e_{k}}$ (note that *k*_1_ ≠ *k*_2_)

o If Lt(*M*_*ij*_) = Rt(*M*_*ij*_) = *k*, then *M*_*ij*_ ← *e*_*k*_.

o $S_{e_{k}}\to S_{k}$

o *M*_*ij*_ ← *k*.

o $S_{w_{k_{1}k_{2}}}^{X}\to S_{k}$

o If ${\tilde{M}}_{\text{ij}}$ is numeric, then $M_{\text{ij}}\leftarrow \tilde{V}({\tilde{M}}_{\text{ij}}).$

To estimate the running time of the algorithm, we classify the transitions as *intra-transitions* and *inter-transitions*, depending on whether *M* or $\tilde{M}$ is used respectively. Thus $S_{w_{k_{1}k_{2}}}^{X}\to S_{k}$ is the only inter-transition. The above transitions are applied to the elements of *M*^*a*^ and *M*^*b*^ till no more transition is applicable. This final form of matrix is written as *F*(*M*^*a*^) or simply *F*^*a*^ (similarly, *F*(*M*^*b*^) or simply *F*^*b*^).

**Figure 2:**
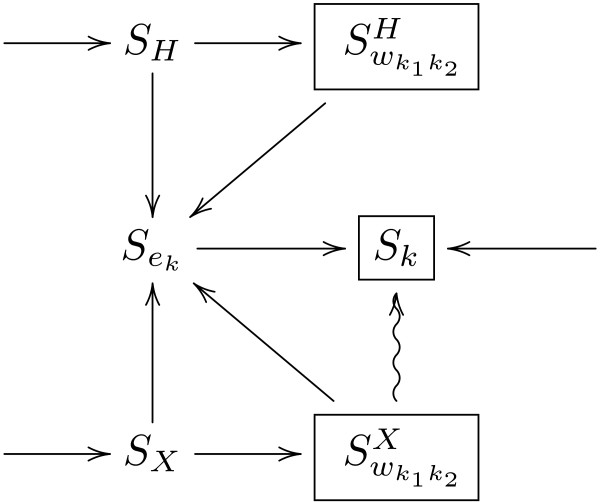
**Transition diagram for computing the final phasing output.** The diagram shows the permissible state transitions for computing the phasing result matrices *F*. The states *S* are discussed in detail in Methods.

The permissible transitions are captured in the transition diagram in Figure 2. The three possible start states are *S*_*H*_, *S*_*X*_ and *S*_*k*_ and the three possible final states are shown boxed. The curly arrow represents the inter-transition while the straight arrows represent the intra-transitions. Note that each *M*_*ij*_ undergoes no more than three transitions since there are no cycles in the state transition diagram. Also, each state with multiple outgoing edges have non-overlapping conditions, leading to a unique choice of transition.

Each element of *F*^*p*^ is in the final state, i.e., for each position 〈*ij*〉, $F_{\text{ij}}^{p}\in \{0,1,w_{01},w_{10},-1\}$, *p* = *a*,*b*. A numeric value of -1 indicates that no information regarding the haplotypes can be deduced. It can be verified that these state transitions induce the following properties on *F* and *V*.

###### 

**Observation 5.** *For a fixed i and p = a,b, the following hold: *

(1) If ${\tilde{F}}_{\text{ij}}^{p}$ is not numeric but $F_{\text{ij}}^{p}$ is, then $V_{j}^{p}(0)=V_{j}^{p}(1)$ must hold.

(2) If ${\tilde{F}}_{\text{ij}}^{p}$ and $F_{\text{ij}}^{p}$ are both not numeric, then 〈*ij*〉, must be a heterozygous trio. The converse however is not true.

(3) If $F_{\text{ij}}^{p}=$ -1, for some *j*, then $F_{\text{ij}}^{p}={\tilde{F}}_{\text{ij}}^{p}=$ -1, for all *j*.

###### 

Observation 6.

(1) Given *M*^*a*^ and *M*^*b*^, *F*^*a*^ and *F*^*b*^ are unique.

(2) *F*^*a*^ and *F*^*b*^ can be constructed from *M*^*a*^ and *M*^*b*^ in O(mn) time.

To show that *F*^*a*^ and *F*^*b*^ are unique, the iterations can be viewed as of two types: one where only inter-transitions and the other where only intra-transitions are applicable. In the very first iteration, due to the possible start states, no inter-transition can be applied. Thus using uniquely applicable intra-transitions, each $M_{\text{ij}}^{a}$ and each $M_{\text{ij}}^{b}$ is transformed. When no intra-transition can be applied, the uniquely applicable inter-transitions are applied. Thus the iterations alternate between intra- and inter-transitions. Hence the final forms *F*^*a*^ and *F*^*b*^ are unique. Next, since each entry can go through no more than three transitions, it is possible to obtain *F*^*a*^ and *F*^*b*^ from *M*^*a*^ and *M*^*b*^ in O(mn) time using suitable list data structures.

#### ***Phase III: Staging output***

An optimization problem (e.g., minimizing an appropriate error function) whose solution is associated with an output configuration, such as alignment of multiple sequences or a phylogeny topology or landscape of crossovers in chromosomes, has the added burden of proving stability in the solution. In other words, how distinct in configuration are the next closest solutions? This is usually very difficult to answer, and most methods are inadequate in addressing this. However, due to the very specific nature of our problem, we provide an agglomerate of “best” solutions, so that its stability can be gauged. Our focus is not just on *a* maximum likely or *a* most parsimonious solution, but on an “agglomerate” of *all* (equally-likely) feasible solutions. This is a characteristic not just of the method but, in a sense, that of the data as well.

Suppose there are *L* > 0 feasible distinct solutions for a given . Is it possible to generate an agglomerate in a single pair of matrices *R*^*a*^ and *R*^*b*^ that captures all the *L* solutions ? In the following paragraphs we describe how to construct the agglomerate (*R*^*a*^ and *R*^*b*^) based on the encodings in *F*^*a*^ and *F*^*b*^.

Let the conjugate of *F* be $\tilde{F}$ defined as ${\tilde{F}}^{a}=F^{b}$ and ${\tilde{F}}^{b}=F^{a}$.d $R_{\text{ij}}^{p}$ that encodes all the possible solutions, is constructed from $F_{\text{ij}}^{p}$, *p* = *a*,*b*, as follows. We use new symbols ∗, *q* and *Q* in addition to the numeric values (0 and 1) in *R*^*p*^. For each progeny *i* and *p* = *a*,*b*: 

1. If $F_{\text{ij}}^{p}=-1$ for some *j*, then without loss of generality $R_{\text{ij}}^{p}\leftarrow 0$ and ${\tilde{R}}_{\text{ij}}^{p}\leftarrow 1$, for all *j*.

2. If $F_{\text{ij}}^{p}\in \{0,1\},$ then $R_{\text{ij}}^{p}\leftarrow F_{\text{ij}}^{p}.$

3. For each *j*, if ${\tilde{F}}_{\text{ij}}^{p}$ is numeric and $F_{\text{ij}}^{p}$ is not, then $R_{\text{ij}}^{p}\leftarrow q$.

4. For each *j*, where $F_{\text{ij}}^{a}$ and $F_{\text{ij}}^{b}$ are both not numeric, then

(3) $$R_{\text{ij}}^{a},R_{\text{ij}}^{b}\leftarrow \left\{\begin{array}{ll} {}* & \text{if at least 1 parent homozygous at}\;j\\ q & \text{if no parent homozygous at}\;j\;\text{with}V_{j}^{p}(1)={\tilde{V}}_{j}^{p}(0)\;\text{\&}F_{\text{ij}}^{p}\neq {\tilde{F}}_{\text{ij}}^{p},\\ q & \text{if no parent homozygous at}\;j\text{with}\;V_{j}^{p}(1)={\tilde{V}}_{j}^{p}(1)\;\text{\&}F_{\text{ij}}^{p}={\tilde{F}}_{\text{ij}}^{p},\\ Q & \text{otherwise.} \end{array}\right)$$

This is illustrated in the following example.

##### 

**Example 1.** *Let *$V_{j}^{a}(0)=V_{j}^{b}(1)=\text{A}$*and *$V_{j}^{a}(1)=V_{j}^{b}(0)=\text{C}$*, for some j. Further let *$F_{\text{ij}}^{a}=w_{10}$*and *$F_{\text{ij}}^{b}=w_{01}$*for some i. Then by Equation 4, *$R_{\text{ij}}^{a}=R_{\text{ij}}^{b}=Q$*. However if *$F_{\text{ij}}^{a}=F_{\text{ij}}^{b}$*, then *$R_{\text{ij}}^{a}=R_{\text{ij}}^{b}=Q$*.*

The non-numeric values in *R*^*p*^ encode multiple solutions, possibly with multiple crossovers. Hence we call the contiguous blocks of non-numeric values as *dispersion intervals*. The details of interpreting these intervals is discussed in the following sections.

### Precision measures of iXora output

Note that *d*_*c*_ is the number crossovers seen in the result matrices, thus an average number of crossovers for the individuals is *d*_*c*_/*n*. But these also correspond to an output configuration. Then how precise is the output of iXora for an input genotype matrix ?

The matrices *R*^*a*^ and *R*^*b*^ enable the elicitation of the parameters for the various haplotype distributions across the markers as well as the precision measures. The agglomerate structure aids in defining a stability measure summarized as the metric $\Delta_{I}$ (Equation 11). The other inadequacies, such as insufficient information and errors due to imperfect experiments or data-acquisition, are evaluated by the methodology and summarized as $D_{I}$ and $E_{I}$ respectively (Equations 12-13). The former is the extent of dispersion in position of each crossover over the agglomerate, and the latter is the observed error in the *R* matrices. The trio define the haplotype precision in the given genotypes.

#### ***Stability measure,*** $\Delta_{I}$

Let *r* be a run of contiguous values in a row (progeny) of *R*^*p*^. A dispersion interval is a non-numeric run, sandwiched by the chromosome boundary or numeric value. Let *R*^*a*^ and *R*^*b*^ be runs in the two haplotypes of the progeny and are aligned in the examples discussed below. It is possible that only one of *R*^*a*^ or *R*^*b*^ is a numeric run. Then it is called a *asymmetric*. But if both are non-numeric, then by construction (Equation 4) *r*^*a*^ = *r*^*b*^ and the run is *symmetric*. A *switch* is defined to occur between *q* and *Q* or between *Q* and the numeric value that sandwiches the run. The number of switches is written as *sw*(*r*). The value ∗ is a wild card and can be treated either as *q* or *Q*. The switch detection is succinctly described by the following two examples. In the illustrations the dispersion interval is shown in green sandwiched between numeric values (in black) and each switch is marked as a numbered (red) down-arrow.

##### 

**Example 2.** *Consider the following run. *

The number of switches is 4 as shown. 

The wild card may result in different positions of the same switch corresponding to whether it was interpreted as a *q* or a *Q*. These distinct positions of the switch is termed the *wild card count* of a switch. If a switch position is not affected by any wild card, its count is 1.

##### 

**Example 3.** *The following run has three wild cards.*

The first is treated as a *q*, the second as a *Q* and the third can be treated as a *q* or *Q* with two possible positions for the third switch. 

The wild card counts of the four switches are 1, 1, 2 and 1 respectively.

When *sw*(*r*) > 0, the location of the switches may define additional positions 〈*ij*〉 in *R* that can updated from non-numeric to numeric. These are the positions that are to the left of the leftmost switch and to the right of the rightmost switch positions. Thus in the run of Example 2, the two *q*’s on the left cannot take the value 1, hence they can be assigned 0, thus the length of the dispersion interval shrinks from 8 to 6. 

Similarly the length of the dispersion interval of Example 3 shrinks from 11 to 9. 

The transformed values are shown in bold above. The same process is applied to every dispersion interval to transform the matrices *R*^*a*^ and *R*^*b*^. The observations from the transformations above is summarized as:

##### 

**Observation 7.** *R is such that for each dispersion interval r, if s w(r) > 0, then (1) r begins and ends with Q and (2) the positions of the first and the last switch sandwich the dispersion interval.*

In fact, the following is easily verified:

##### 

**Observation 8.** *Let r be a dispersion interval and let s = s w(r). Then (1) the total number of crossovers in the interval, across both the haplotypes, is exactly s and each crossover in each haplotype of the configuration is at a position of the switch.(2) If r is asymmetric, then s is zero. In general, s is even and the number of crossovers in each haplotype of each distinct configuration is odd.*

In practice, we observed that in all data sets all the dispersion intervals had no switches. There was exactly one instance where *sw*(*r*) was 2. Also, the following is easily verified.

##### 

**Observation 9.** Let *s* = *sw*(*r*) for a dispersion interval *r*. Then if *s* ≤ 2, there is no additional crossover introduced by *r*. If *s* > 2, the number of additional crossovers is *s*-2.

Let *U* be the set of all dispersion intervals in *R*. Then

(4) $$\Delta_{I}=\underset{r\in U,\text{sw}(r)>2}{\sum }\left(\text{sw}(r)-2\right).$$

#### ***Dispersion index,*** ${\text{D}}_{I}$

The dispersion index, $D_{I}$, is a measure of the uncertainty in the position of the crossovers. This is computed as an average over all predicted crossovers. Thus

(5) $$D_{I}=\frac{\underset{r\in U}{\sum }\delta (r)}{\left(m-1)(d_{c}+\Delta_{I}\right)},$$

with

$$\begin{array}{lll} \delta (r) & =\left\{\begin{array}{l} \text{len}(r)\;\;\text{if}\;\;\text{sw}(r)=0\\ \underset{l}{\sum }2c_{l}\begin{array}{l} \text{otherwise} \end{array} \end{array},\right)\; & \end{array}$$

where *c*_*l*_ is the wild card count of each switch *l* in *r*. When the location of each crossover is known precisely, this index is 0, while value 1 indicates maximum uncertainty in the location.

#### ***Error estimate,*** $E_{I}$

If $R_{\text{ij}}^{a},R_{\text{ij}}^{b}\in \{0,1\},$ then the position 〈*ij*〉 has a *mismatch* if $\{V^{a}(R_{\text{ij}}^{a}),V^{b}(R_{\text{ij}}^{b})\}\neq I_{\text{ij}}.$ Note that the mismatch at this position could be flagged as an error or one of $R_{\text{ij}}^{a}$ and $R_{\text{ij}}^{b}$ can be modified to potentially introduce additional crossover(s). These are undetectable during the lower bound computation and do not overlap with the dispersion intervals. Let *N* be the number of such mismatches. Then, $E_{I}$, the error estimate in  is defined as

(6) $$E_{I}=N/\mathrm{mn.}$$

Also, in our experiments these mismatches were extremely low (less than 0.01) and when followed up turned out to be experimental errors. Hence we have followed the convention that such a mismatch be flagged as a potential error. Then an error, if any, is at 〈*ij*〉 in *F* that underwent the transitions $S_{X}\to \cdots S_{e_{k}}\to S_{k}$. Note that the converse is not true. Also, an additional crossover, if any, is at 〈*ij*〉 in *F* that underwent the transitions $S_{X}\to S_{w_{k_{1}k2}}^{X}$. Again, the converse is not true.

To summarize, the trio ($\Delta_{I},D_{I},E_{I}$) define the haplotype precision in the given genotypes . Across our experiments (30 distinct data sets) with real data [15], the mean precision trio were observed to be (0,0.002165,0.000299).

### Downstream processing of iXora output

Since iXora associates the parent haplotypes (not just the inherited alleles) to each marker in a progeny, it is possible to study the distributions of the inherited parent haplotypes independent of or in association with a phenotype. The details of these and other related postprocessing available in the iXora framework are described here.

#### ***Haplotype frequency distributions***

One of the important consequences of haplotype inferencing is obtaining the haplotype frequency distribution across the chromosomes. A marker *j* of progeny *i* has two alleles, one derived either from haplotype 0 or haplotype 1 of parent *a* and the other either from haplotype 0 or haplotype 1 of parent *b*. Since *R* is an agglomerate, it also contains the non-numeric values that encode multiple configurations. Based on the encoding in *R* we estimate the expected value of the frequency count and its variance. First, we enumerate the feasible solutions encoded by a non-numeric run *r*.

##### 

**Example 4.** *In each of the following the length of the dispersion interval is 3 and the number of additional crossovers is zero. However, the number of configurations are different based on the structure of the switches. Two runs with zero switches in each:
*

A run with *sw*(*r*) = 2: 

The 8 distinct configurations for Example 2 are: 

Let *wt*(*r*) be the number of distinct solutions encoded by *r*.

##### 

**Observation 10.** *Let s = s w(r). Let c*_1_*,c*_2_*,…c*_*s*_ *be the wild card counts of the switch positions. Let w t(r) be the number of distinct feasible solutions due to r. Then*

(1) If  < 2, w t(r) = l e n(r) + 1.

*(2) If s  ≥ 2, *$\text{wt}(r)=Z(r)\overset{s}{\underset{l=1}{\prod }}c_{l}$*, where *

(7) Z(r)=∑l=1⌈s/4⌉-1s2l-1+s2⌈s/4⌉-1.

(1) above is easily verified and for (2), Equation 17 is explained through the following example.

##### 

**Example 5.** *Consider the following r with s w(r) = 6. *

Note that the 6 switches can be shared by the two haplotypes as 1 and 5 (the first column), or as 3 and 3 (second column) as follows.


*There are six distinct configurations for the first case and since the two haplotypes can be switched it gives 2 × 6 distinct configurations. For the second there are*63=20*distinct configuration, giving a total of 12+20 distinct solutions due to r*.

#### ***Expected frequency and variance***

Based on *R*, we estimate the expected value of the frequency count of the haplotype pairs and its variance. If *R*_*ij*_ is non-numeric, let *R*_*ij*_ = *α* hold. Thus for each marker *j* consider the following, for *x*,*y* = 0,1,*α*,

$$c_{\text{xy}}=|\left\{i|R_{\text{ij}}^{a}=x\;\;\text{and}\;\;R_{\text{ij}}^{b}=y\right\}|$$

In the absence of any other external information, each of the alternative solutions in *R* is equally likely. Under this assumption, it is easy to verify the following:

##### 

**Observation 11.** *The expected count, *${ĉ}_{k_{1}k_{2}}$*, of each haplotype pair, k*_1_*,k*_2_ = 0,1, is:

$$\begin{array}{lll} {ĉ}_{k_{1}k_{2}} & =c_{k_{1}k_{2}}+\Delta_{k_{1}k_{2}},\text{where}\; & \;\\ \Delta_{k_{1}k_{2}} & =c_{\alpha k_{2}}/2+c_{k_{1}\alpha }/2+c_{\alpha \alpha }/4.\; & \end{array}$$

*The variance *$\sigma_{k_{1},k_{2}}^{2}$*of the count of the haplotype pair is approximated as *$\Delta_{k_{1}k_{2}}$.

#### ***Haplotype-phenotype association analysis***

Below, we describe the iXora methodology for associating discrete traits with genomic locations using haplotypes. The same approach can be used for continuous traits, using different statistical tests and randomizations. In general, the phasing output can be used in other types of statistical tests, for example to test for associations between a pair of markers and a phenotype. In the following, let *L* the number of discrete phenotype values.

##### 

**Combination of parents** We can test the effect of each haplotype pair at a marker with a phenotype as follows. In the case of discrete phenotype with *L* possible values, iXora constructs for each marker a 4 × *L* contingency table of haplotype pair & phenotype counts. In the current iXora implementation, we use Fisher’s exact test *fisher.test* in R [17] to test for association between haplotype pairs & phenotype. The test outputs a *p*-value for each marker, denoting the significance of the association.

##### 

**Individual parents** We can also investigate the contribution to phenotype of each parent individually. The contingency table in this case is a 2 × *L* table of haplotype & phenotype counts (a separate table for each parent at each marker). Fisher’s Exact test is used to test for association between haplotypes & phenotype. The test outputs a *p*-value for each marker and for each parent, denoting the significance of the association.

##### 

**Significance thresholds via randomization** We include a method for directly estimating the background distribution of *p*-values in the haplotype-phenotype data by randomizing the phenotype labels. If *p*-values observed in the randomized data are always larger than in the real data, then the findings on real data may indeed be significant. When working with categorical traits, we take into account the size (number of individuals) of each phenotype category in the permutation test. Let *S* be the size of the smallest category. For the permutation test, we randomly select *S* individuals from each category and permute their phenotype values. The permutation test is repeated *T* times, running the same statistical test on the randomized data as on the real data, for each marker. The smallest *p*-value observed in the randomized data is generated as output and becomes the threshold for significance in the real results.

#### ***iXora output visualization***

The agglomerate solution from the phasing algorithm can be directly visualized to detect distortions in the data, with or without using phenotypic information. Approaches for visualizing the phasing solution are demonstrated in the following two paragraphs, while the third paragraph describes visualization of haplotype-phenotype associations. The figures shown here as examples stem from the use case described in detail in the Section “Using iXora” in Additional file 1. Therein is detailed the phasing an trait association process for locating the genomic region and specific haplotype that determines the simulated phenotype (*height*).

##### 

**Individual haplotypes** The individual haplotypes can be directly visualized, for example as the colored haplotype blocks shown in Additional file 1: Figure A3. The visualization indicates the locations of crossovers in each individual, including uncertainty in the crossover locations. As an alternative visualization, Additional file 1: Figure A4 shows iXora output on the frequencies of individual haplotypes per parent. In this case, the individuals are divided into two groups by phenotype, and clear distortion is observed regarding frequency of haplotypes in each group.

##### 

**Figure 3:**
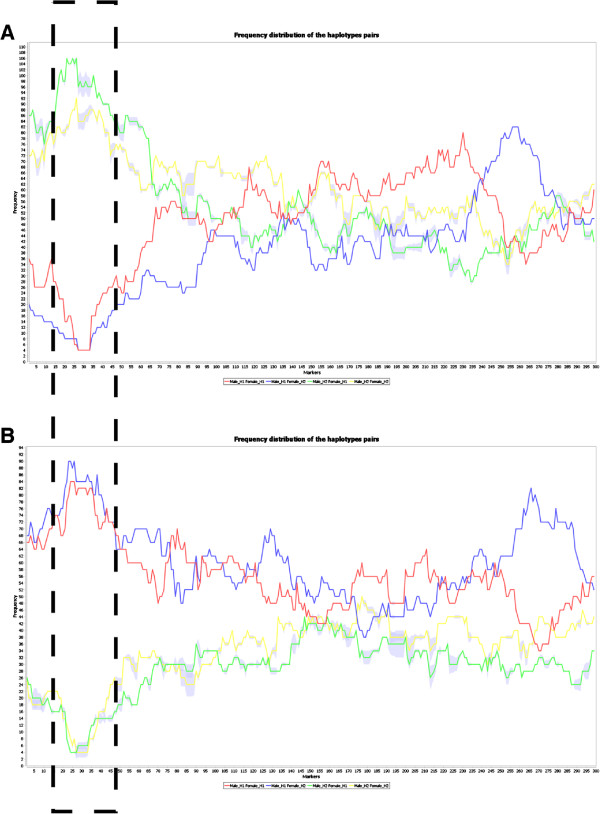
**Expected haplotype distributions visualization.** Expected haplotype frequencies ${ĉ}_{k_{1}k_{2}}$ are shown for the simulated use case detailed in Additional file 1, for the two phenotypic groups: **A**) *tall* progeny, **B**) *short* progeny. The variance $\Delta_{k_{1}k_{2}}$ due to uncertainty in crossover locations is shown as shaded regions. Clear distortion is visible near marker 30 (marked by the dashed rectangle), evident from under-representation of haplotype combinations involving paternal haplotype H2 in the *short* progeny (green and yellow lines in **B**).

**Haplotype pairs** The agglomerate structure capturing all the equally-likely solutions enables estimation of the possible dispersion of the crossover locations. iXora visualization of the expected frequency distributions of the progeny haplotype pairs is shown in Figure 3 for two phenotype groups. Clear under-representation of two distinct haplotype pairs is observed for each group. This visualization can be used to spot unexpected distortions, whose significance can be further evaluated using statistical tests.

##### 

**Figure 4:**
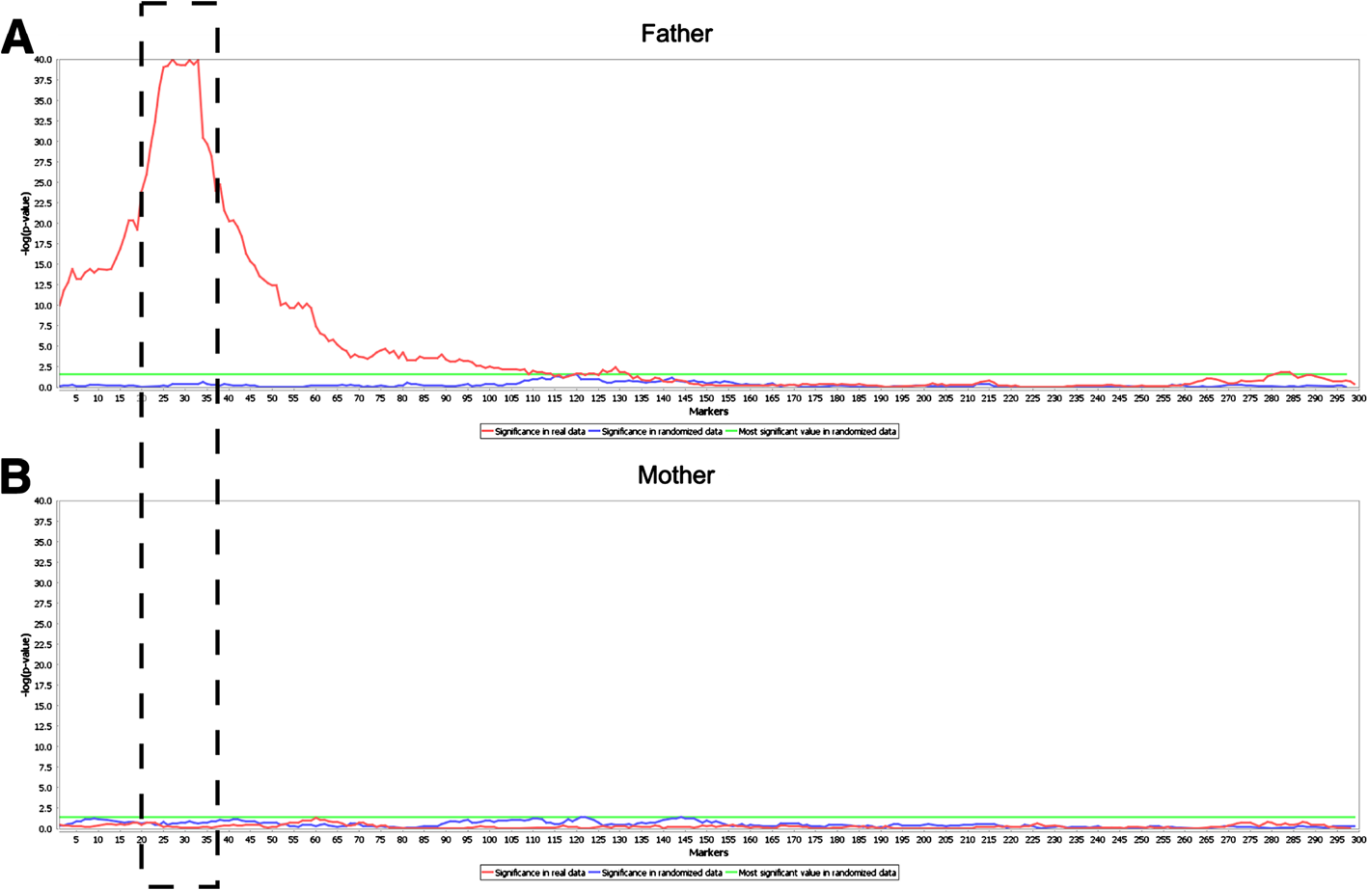
**Results from Fisher’s exact test for phenotype-haplotype association for A**) **Father and B**) **Mother, including the p-value significance thresholds from randomizations, for the simulated use case detailed in Additional file 1.** In this case only one region of the genome from the father is significantly associated with the phenotype (marked by the dashed rectangle), according to the Fisher’s exact test and the randomization thresholds. [Legend: real data (red), randomized data (blue), smallest value in randomized data (green)].

**Phenotype association** Phenotype association for each parent individually is shown in Figure 4. The *p*-value threshold obtained via randomizations is also shown. In this case it is evident that only one parent is associated with the phenotype, in one genomic region. An example of iXora results from statistical testing for haplotype pairs’ association with phenotype is shown in Additional file 1: Figure A5, and a comparison between genotype and haplotype association results is shown in Additional file 1: Figure A6.

### Comparison with related methods

Here we first elaborate on the three distinct categories of population models for phasing, and then give details on the comparison of iXora with related phasing methods in literature. Technical details on the settings for each compared method can be found in Additional file 1.

#### 

##### 

**Unrelated individuals (no parent information)** These methods treat the input genotypes as samples from a population of unrelated individuals, and do not assign the progeny to parental haplotypes. While they may be applicable to human population data, they are not suitable for our purposes. fastPHASE can be adapted to our problem setting by treating the input as *n* + 2 individuals from a population originating from four founder haplotype clusters. We adapt MACH similarly, though it has a much larger default value on the number of haplotypes. FMPH (Integer Programming Formulations To Solve Maximum Parsimony Haplotyping) [12] is closest in spirit to iXora, but is computationally very expensive and suitable only for small data sets (up to ≈50×30), although a hybrid approach is suitable for slightly larger data sets (up to ≈150×100). While the limitation on number of individuals can be tackled by splitting the data (as we do for Merlin, HAPI, SHAPEIT2 below), the limitation on the number of markers is debilitating, so we were unable to run FMPH on our data sets.

##### 

**Unrelated trios** These methods allow the definition of family relationships between parents and progeny in the input, with the limitation that each parent has only one progeny. BEAGLE and HAPI-UR (HAPlotype Inference for UnRelated samples) are two such methods. The methods phase the progeny individually in terms of sequences that were transmitted from each parent. Therefore the progeny are not necessarily assigned to a consistent set of parent haplotypes.

##### 

**Related trios** These methods allow defining several progeny originating from the same two parents, thus the underlying algorithms utilize the full sibling information. However, unlike iXora, none of the existing methods was able to use the entire set of progeny per two parents. In our application this size is in hundreds. HAPI and Merlin produce results only on families of about 10 progeny while SHAPEIT2 can only process sizes up to 50. We therefore randomly divided the progeny into sets of appropriate sizes and phased the sets independently. However, we observed that the phasing results for the parents between sets were often inconsistent, affecting the overall accuracy. HAPI and Merlin produce an assignment of progeny to parental haplotypes while SHAPEIT2 does not.

##### 

**Comparison measures** Here we describe the measures that we used to compare the different methods. Since existing phasing methods generate various types of output, we use two different measures so that all the methods are comparable on at least one measure. Our interest was not simply restricted to phasing the progeny genotypes by assigning each allele to the *correct parent* (PA), but also assigning them to the *correct haplotype of that parent* (PHA).

First, the phasing accuracy (PA) of progeny is examined, on a marker-by-marker basis, of only the heterozygous positions. We report the number of correctly assigned and the unassigned (unknown) positions as a percentage. BEAGLE, HAPI, HAPI-UR, Merlin, SHAPEIT2 and iXora can be directly compared on this measure for progeny, because they report the parental origin (maternal, paternal) of each allele. To incorporate fastPHASE and MACH also in this comparison, we post-processed their output as follows: progeny haplotypes are labeled as ‘maternal’ and ‘paternal’, using the labeling that minimizes mismatches compared to true maternal and paternal haplotypes. After the post-processing, all methods can be compared on this measure for progeny. The same accuracy evaluation is used to report imputation accuracy, by examining only the phasing for the missing input values.

Second, the accuracy of assigning the correct parental haplotype (PHA) for each progeny allele is examined, again on a marker-by-marker basis. *All* markers, including homozygous positions are used. For the output of programs where the input had to be split into smaller families, we consider only those subsets of progeny whose parents’ phasing are consistent with the majority parents (see Additional file 1 for details). Again, we report the number of correctly assigned and unassigned (unknown) positions, deeming a progeny position to be correct only when *both* alleles of the genotype are assigned to the correct parental haplotype. Only HAPI, Merlin, and iXora can be directly compared on this measure for the progeny.

All the simulated data sets are available at the iXora website http://researcher.ibm.com/project/3430.

## Competing interests

The authors declare no competing interests.

## Authors’ contributions

FU and NH designed and implemented the framework. FU designed and implemented the user interface. LP designed and implemented the core iXora algorithm for haplotype extraction. NH and FU designed the experiments and performed the analysis described in this paper. OEC contributed to the comparison with existing phasing methods (fastPHASE, SHAPEIT2). DL and SR were instrumental in the algorithm verification on real (Cacao) data. JCM, RJS and DNK contributed to identifying the scope of the iXora pipeline. LP, NH, and FU wrote the paper. All authors read and approved the final manuscript.

## Supplementary Material

Additional file 1 **Additional text and figures.** The file contains an example on using iXora on a simulated phasing and trait association scenario. Additionally, the file includes visualizations of the iXora framework and user interface. The file also contains technical details on the comparison with related methods. Click here for file
