# Supplementary material for: iXora: exact haplotype inferencing and trait association
Source: BMC Genet. 2013 Jun 6;14:48. doi: 10.1186/1471-2156-14-48 (PMC3716545; doi:10.1186/1471-2156-14-48)
Supplement: Additional file 1 — Additional text and figures. The file contains an example on using iXora on a simulated phasing and trait association scenario. Additionally, the file includes visualizations of the iXora framework and user interface. The file also contains technical details on the comparison with related methods. [file 1471-2156-14-48-S1.pdf]

Additional File 1
# - iXora: Exact Haplotype Inferencing and Trait Association

Using iXora: an example

In this section we first show the various functionalities of iXora by using a simulated data set. Next, we demonstrate that haplotypes (PHA, in particular) yield a higher resolution map of the trait of interest than genotypes.

Outline. The overall framework, described in Figure A1, infers haplotypes from genomic marker data of a population of related individuals, and then uses this to map the genomic regions of interest associated with observed phenotypes of individuals in the population. In particular, it provides for statistical tests (such as Fisher's exact test or chi-squared hypothesis test) on the parental haplotype distributions with trait values for mapping studies, or, without trait values for haplotype distortion studies. To assist the user in ruling out spurious inferences, the framework also provides for built-in randomization procedures. The output from both the stages, phasing and subsequent postprocessing, can be visualized through a user-friendly graphical interface. A screenshot of the interface dialog, including examples of visualizations, is shown in Figure A2. For additional details, the reader is directed to the iXora user manual (http://researcher.ibm.com/project/3430).

Experimental set-up. We simulate a pair of heterozygous parents and progeny with 300 markers placed at uniform 1 cM genetic distances. We generate a population of 200 progeny by simulating crossovers, based on the Haldane model [1], at a rate of 0.01 probability of crossover between adjacent markers in each gamete. As a result, on average 1% of the progeny haploids have a crossover between any two adjacent markers. The parental haplotypes were constructed by mimicking our observations from real data, having equal proportions of homozygous and heterozygous sites. Haplotype inference is performed separately for each chromosome on real data; this example represents the analysis of a single chromosome.

The phenotype of *height*, which has values (*tall* and *short*), is simulated as follows. For simplicity, let *height* be a mono-genic trait with two alleles G_1 and G_2. Let G_2 be dominant for *tall* and G_1 be recessive. Also let this gene be located between markers 30 and 31. Further, the father has the genotype (G_1,G_2) and the mother (G_1,G_1); by definition, the father is *tall* and the mother is *short*. Similarly, the phenotype is associated with each of the progeny that are tracking the inheritance of this gene.

We mask at random 5% of the total genotype values of the progeny and the parents, so as to simulate missing genotyping data. In addition, to simulate phenotyping errors, we assign an incorrect phenotype for

5% of the progeny. Following this process, we have 107 *tall* and 93 *short* progeny.

Haplotype extraction. iXora haplotype inference was applied on the simulated genotypes. The parental haplotypes for 23 markers with at least one missing parental genotype were successfully resolved. However, there were six markers (with exactly one parental genotype missing) that remained unresolved. The phasing was thus able to utilize 98% of the markers (i.e., 294 out of the 300), even when 5% of the total genotypes were missing, including the parental genotypes.

The error measures on the phasing result indicate that the result is very precise: the stability measure is obtained ($\Delta = 0$), the crossover positions are defined precisely ($D = 0.00937$), and the effective error in the data is negligible ($E = 0.000149$) (see Methods for details on the measures).

In Figure A3, the phased haplotype frequencies are visualized as fragments from the parental haplotypes. This overall picture shows that crossovers are uniformly distributed across the genome, and that the uncertainty (shown in green) is contained in very short segments.

Phenotype association. The haplotype frequencies, when the progeny are categorized by phenotype, are shown in Figure A4. The expected frequencies of haplotype pairs for each phenotypic group are visualized in Figure 3 in the main manuscript. From these figures it becomes clear that the correct region, around marker 30, is identified as having clear haplotype distortion when considered with the phenotype classification.

The results from Fisher's exact test confirm the visual observations; the smallest p-value is observed at marker 28 (for the combination of parents and for the father); whereas the entire region, from marker 26 to 34, is observed to be very significant. The randomization test, which was repeated 100 times, confirms that this is in fact the only region in which the genomic data is significantly associated with the phenotype. These results are visualized in Figure A5. Additionally, the effect of each parent is separately visualized in Figure 4 in the main manuscript.

Based on the haplotype distortion plots, we can infer that the paternal haplotype H2 is under-represented in *short* individuals. Thus iXora correctly discovers the dominant haplotype for the phenotype *tall*. Despite a number of missing data and artificially perturbed phenotypes, the genomic location associated with the phenotype was accurately mapped within a resolution of a few markers.

Note that it is possible to apply the statistical test, as described in the last sections, independently to sets of $k > 1$ markers to study k-way interactions. Indeed, an appropriate value of k depends on the number of individuals in the population ($k \ll$ number of individuals, in order to obtain statistically meaningful results).

Although the statistical association test was applied independently at each marker locus, as discussed in the last sections, we still observe an improvement in accuracy, such as in peak contrasts in Figure A6 or in significance thresholds via randomizations (results not shown), as well as improvement in the mapping resolution through the use of haplotypes rather than genotypes. Why does the haplotype information work better than the genotype? We explore this question here.

Increased accuracy with haplotypes. Haplotyping clusters the individuals into a larger number of groups, which are also more biologically relevant. As an example, let marker j be bi-allelic, with values `A` and `T`. Genotype information then partitions the individuals of the population into at most three groups defined by the values `AA`, `TT` and `AT`. In contrast, the haplotype information partitions the populations into at most four groups, which are defined by `F1M1`, `F1M2`, `F2M1` and `F2M2`, where `Fi` is the `i`th haplotype of the father, `Mi` is the `i`th haplotype of the mother, for `i = 1,2`. Furthermore, this partition is biologically more meaningful as well, because the parental inheritance of the markers (in the haplotypes) usually plays a significant role in determining the traits of the individual. Thus this refined partition, with biological relevance, contributes to an increased accuracy of the associated genomic region. Another additional factor is the successful imputation of many loci with missing genotypes by iXora.

Increased mapping resolution with haplotypes. Note that when a locus is homozygous in both the parents, say `AA` and `TT` respectively, then the progeny are identical, i.e., `AT`, at that locus. Thus a statistical association test on the genotypes is not applicable at that locus. On the other hand, iXora resolves most, if not all, of these positions in the process of haplotyping. Thus, the statistical tests continue to be applicable to the haplotypes at this locus as well, thereby contributing to increased resolution in the results.

Back to the example of the last section. A genotype-based test would only be able to utilize 221 (75%) of the markers, because in the remaining 25% of the markers, the genotypes of all progeny are identical. In the first step of phasing, 98% of the markers, irrespective of the identical progeny values at some of the markers, were utilized. Additionally, iXora imputes missing genotypes in the progeny in the haplotype extraction process (based on minimizing the overall number of crossovers in the population). Therefore a larger proportion of the input data is usable in the analysis of the second step.

We next compare results of statistical tests on genotype information with results on haplotype information. The results of a genotype-based Fisher's test are shown in Figure A6. The haplotype test, unlike the genotype test, demonstrates the entire region between markers 26 and 34 to be significantly associated with the phenotype. In contrast, the genotype test shows a peak at markers 23 and 35 while the marker of interest and nearby markers appear not to be associated with the phenotype. We find that the parents are

in fact homozygous at the markers in this region. However, the haplotype extraction is unaffected by this, and correctly maps the phenotype. Additionally, the test on phased data is able to identify the parental haplotype (paternal H2) that is consistently associated with the trait in the region of interest.
Furthermore, the region between the two peaks in the genotype test spans several markers and may be a large genomic region, which could lead to a possible hypothesis that the two peaks represent two separate loci that are associated with the trait.

Technical details on the comparison with related methods

All methods were run with default parameters unless otherwise specified in the following. The parameters for the programs that were influenced by the input scenario were chosen as follows while the rest were used with their default values. fastPHASE was run using the "minimize switch error" mode, with 20 random starts for the EM algorithm, iterated 30 times ("minimize individual error" mode gave similar results, data not shown). Merlin was run using the "estimate most likely pattern of gene flow" mode. HAPI was run using the "minimum-recombinant" mode.
In order to handle the large size of the data set, the input for HAPI and Merlin has to be defined as several families with a small number of progeny each. We used ten progeny per family so that the programs generated non-empty result files. Note that for a larger number of progeny the programs produce empty files. The programs produced inconsistent results for the parents for the different families, therefore we apply a *majority rule* to define the parental phasing. We extracted the phasing of the parents that was consistent between the largest number of families, and used this as the majority parents. While it is possible to use other heuristics (such as average) when the phasing for the parents are inconsistent, we chose the majority rule for two reasons. We believe that this would be preferred by users attempting to map traits to markers precisely and thus report the positions in the progeny as "unassigned" that correspond to non-majority parental haplotypes. Also, this leads to an unambiguous definition for the second measure. For the comparisons, iXora results were simply post-processed by assigning a numerical value to any non-numerical flanked by two identical numerical values.

References

1. Lynch M, Walsh B: *Genetics and Analysis of Quantitative Traits*. Sinauer Associates; 1998.

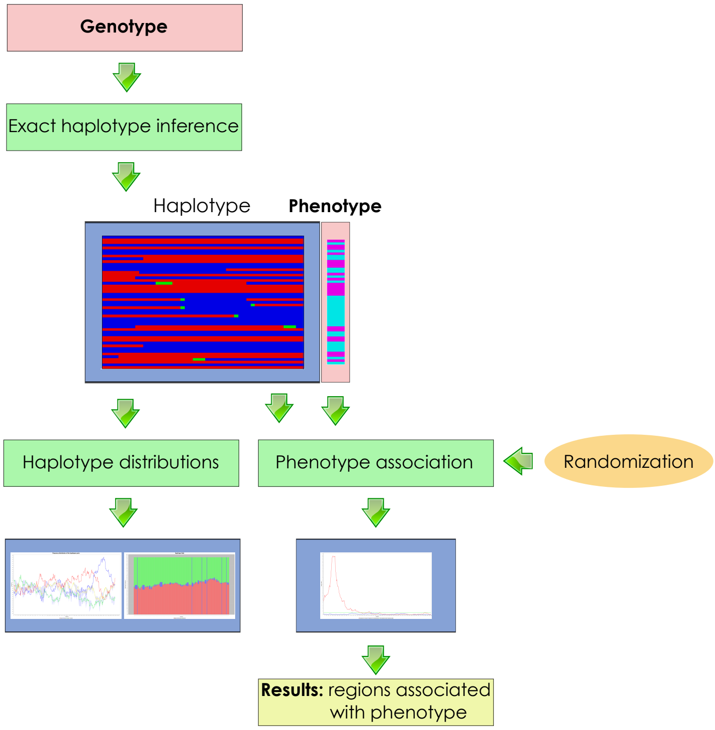

Figure A1: The iXora framework is shown as a flowchart starting from genotype and phenotype data. Haplotypes for several individuals are shown as mosaics from two parental haplotypes (red and blue) as computed by iXora. A phenotype value is associated with each individual of the population.

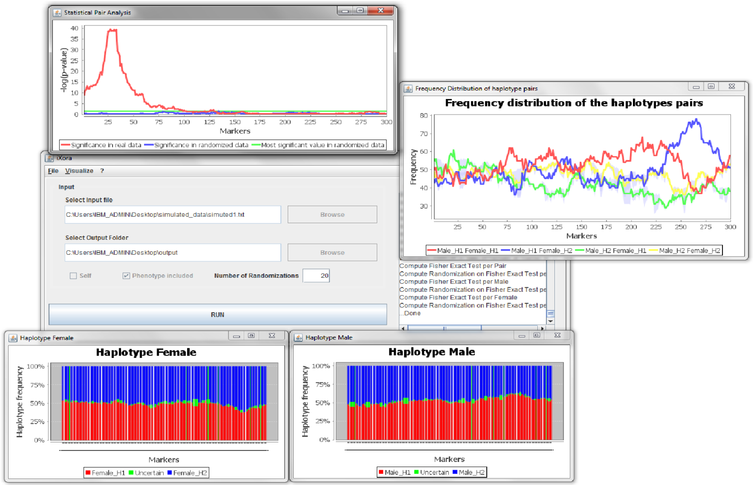

Figure A2: A screenshot showing the main dialog window and visualizations of the phased genotype data.

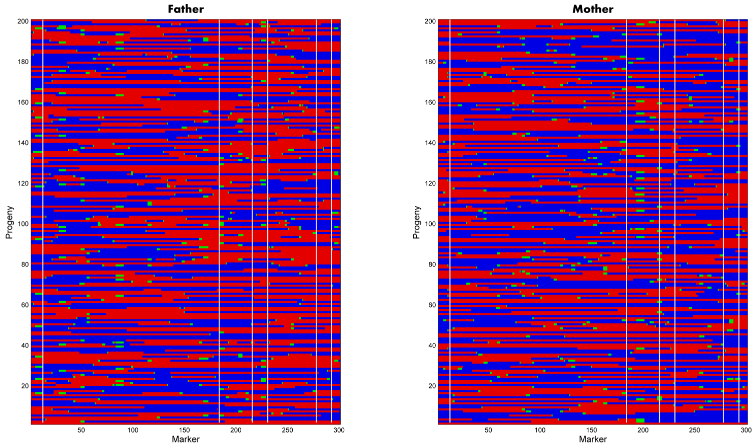

Figure A3: Haplotype frequencies are shown for the simulated case study, for the haplotypes inherited from the father (left) and mother (right). Red denotes haplotype H1 from the parent, blue haplotype H2, while green represents uncertainty in the phasing result (i.e. the crossover location is resolved to an interval, but not a point (marker position)). White represents the markers where a consistent phasing was not obtained.

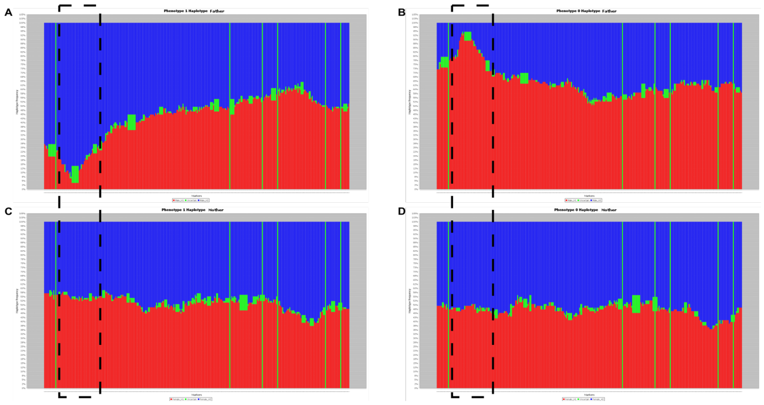

Figure A4: Haplotype frequencies are shown for the simulated case study, after dividing the individuals into two groups according to phenotype: A) Father for *tall* progeny, B) Father for *short* progeny, C) Mother for *tall* progeny, D) Mother for *short* progeny. Red denotes haplotype H1 and blue haplotype H2, while green represents uncertainty. Six unresolved markers are visible as green columns. A clear distortion around marker 30 (marked by the dashed rectangle) can be observed regarding frequency of the haplotypes between the two groups for the father. For the mother there is no clear distortion.

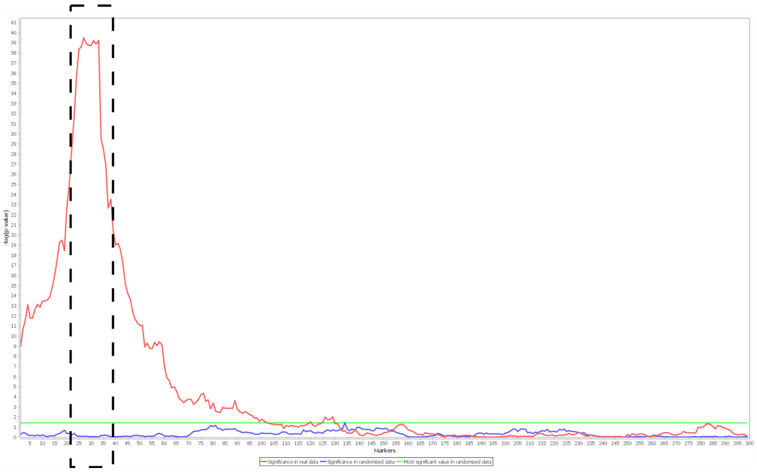

Figure A5: Results from Fisher's exact test for phenotype-haplotype association for pairs of haplotypes, including the p-value significance threshold from randomization. In this case only one region of the genome is significantly associated with the phenotype (marked by the dashed rectangle), according to the Fisher's exact test and the randomization threshold. [Legend: real data (red), randomized data (blue), smallest value in randomized data (green)].

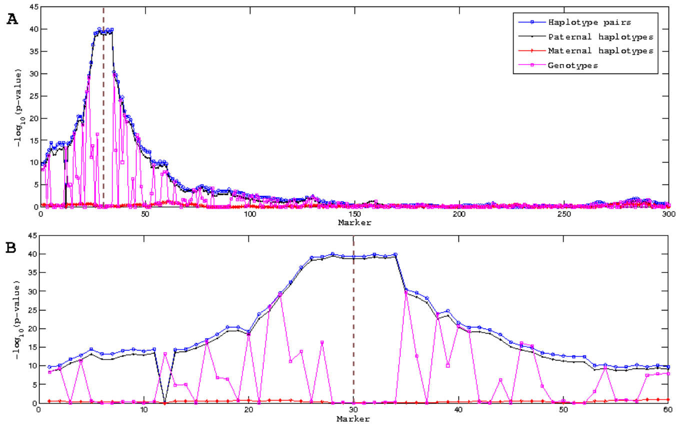

Figure A6: Results from Fisher's exact test for phenotype association using haplotypes, and as an alternative approach, using genotypes are shown. Marker 30, ground truth for association with the phenotype, is shown with a dashed vertical line in A) Entire chromosome, and B) The relevant region. The haplotype-based test clearly shows the relevant region as an extremely significant block, while the genotype-based test shows inconsistent results within the relevant region.
